# Draft Genome Sequence of a Plant Growth-Promoting Rhizobacterium, *Serratia fonticola* Strain AU-P3(3)

**DOI:** 10.1128/genomeA.00946-13

**Published:** 2013-11-14

**Authors:** Usha Devi, Indu Khatri, Navinder Kumar, Lalit Kumar, Deepak Sharma, Srikrishna Subramanian, Adesh K. Saini

**Affiliations:** Shoolini University of Biotechnology and Management Sciences, Department of Biotechnology, Bajhol, Solan, Indiaa; CSIR-Institute of Microbial Technology, Sector 39A, Chandigarh, Indiab

## Abstract

Plant growth-promoting rhizobacteria (PGPR), found in the rhizospheric region of plants, not only suppress plant disease, but also directly improve plant health by improving the availability of nutrients and by providing phytostimulants. Herein, we report the high-quality genome sequence of *Serratia fonticola* strain AU-P3(3), a PGPR of the pea plant, which confers phosphate solubilization, indole-3-acetic acid production, ammonia production, hydrogen cyanide (HCN) production, and siderophore production and also confers activity against *Rhizoctonia* species. The 5.02-Mb genome sequence contains genes related to plant growth promotion and biocontrol activities.

## GENOME ANNOUNCEMENT

The rhizosphere, the layer of soil under the influence of roots, is richer in bacteria than the surrounding soil (1). Many of the microbes present in the rhizosphere work symbiotically to improve plant growth and, thus, are referred to as plant growth-promoting rhizobacteria (PGPR) (1). PGPR exhibit biofertilization and rhizoremediation, stimulate growth of roots, control plant stresses, and reduce plant diseases to augment plant growth (1). Many members of the *Serratia* genus have been shown to confer PGPR traits, including solubilization of inorganic phosphate (2) and phytohormone production and phytoremediation (3), and protect plants from flood-induced damage (4). They also improve plant health indirectly by reducing plant pathogens of bacterial, fungal, and nematodal origin (3, 5–8). We isolated *Serratia fonticola* AU-P3(3), a Gram-negative motile rod from the rhizosphere of pea roots, which confers activity against *Rhizoctonia* species (U. Devi, I. Khatri, N. Kumar, L. Kumar, D. Sharma, S. Subramanian, and A. K. Saini, unpublished results), fungal plant pathogens of the pea (9). AU-P3(3) produces HCN and siderophores that may account for its antifungal activity. AU-P3(3) also produces ammonia and indole-3-acetic acid and exhibits phosphate solubilization (Devi et al., unpublished). Importantly, AU-P3(3) also utilizes insecticides and fungicides as carbon sources, suggesting its role in bioremediation (L. Kumar, U. Devi, I. Khatri, N. Kumar, D. Sharma, S. Subramanian, and A. K. Saini, unpublished results).

Considering the broader biotechnological application of *S. fonticola* AU-P3(3), we went on to sequence its genome. The genome of *S. fonticola* AU-P3(3) was sequenced using the Illumina-HiSeq 1000 technology. Sequencing resulted in 34,600,774 paired-end reads (insert size of 350 bp) of 101 bp. A total of 34,322,211 high-quality reads with approximately 690× coverage were assembled with CLCbio wb6 (word size 35 and bubble size 55) to obtain 49 contigs (*N*_50_, 318,463 bp). The genome finishing module of CLCbio, followed by SSPACE v2.0 scaffolder (10) and GapFiller v1-10 (11), was used. The gap-filled scaffolds thus obtained were broken at the gaps to obtain 44 contigs (*N*_50_, 318,901 bp) of 5,023,127 bp, with an average G+C content of 54%. The functional annotation was carried out by RAST (Rapid Annotations using Subsystems Technology) (12), tRNA was predicted by tRNAscan-SE 1.23 (13), and rRNA genes were predicted by RNAmmer 1.2 (14). The genome contains 3 rRNA genes (5S, 23S, and 16S) and 72 aminoacyl-tRNA synthetase genes. A total of 4,483 coding regions (2,340 genes transcribed from the positive strand and 2,143 from the negative strand) were found in the genome, of which 3,669 (82%) were functionally annotated. The genome coding density is 85%, with an average gene length of 923 bp. The annotated genome has 78 genes responsible for motility and chemotaxis, including 14 genes for flagellar motility. Fifty-three genes are responsible for phosphorus metabolism. Twenty-one genes are osmotic stress response genes, including 5 for osmoregulation and 53 for oxidative stress, to make a total of 139 genes responsible for stress response in this organism.

The functional comparison of the genome sequences available on the RAST server revealed the closest neighbor of *S. fonticola* AU-P3(3) to be *Serratia odorifera* 4Rx13 (score, 502), followed by *S. proteamaculans* 568 (score, 490), *S. odorifera* DSM 4582 (score, 489), and *S. marcescens* Db11 (score, 464).

### Nucleotide sequence accession numbers.

This whole-genome shotgun project has been deposited at DDBJ/EMBL/GenBank under the accession number ASZB00000000. The version described in this paper is the first version, ASZB01000000.
